# The Effect of MSCs Derived from the Human Umbilical Cord Transduced by Fibroblast Growth Factor-20 on Parkinson's Disease

**DOI:** 10.1155/2016/5016768

**Published:** 2016-05-05

**Authors:** Li Jinfeng, Wang Yunliang, Liu Xinshan, Wang Shanshan, Xu Chunyang, Xue Peng, Yang Xiaopeng, Xu Zhixiu, Yin Honglei, Cao Xia, Duan Haifeng, Cao Bingzhen

**Affiliations:** ^1^Neurology Department, General Hospital of Jinan Military Region, Jinan, Shandong 250031, China; ^2^Neurology Department, The Second Hospital Affiliated to Zhengzhou University, Zhengzhou, Henan 450014, China; ^3^Neurology Department, The 148th Hospital, Zibo, Shandong 255300, China; ^4^Sanbo Brain Hospital, Capital Medical University, Haidian District, Beijing 100093, China; ^5^Department of Experimental Hematology, Beijing Institute of Radiation Medicine, 27 Taiping Road, Beijing 100850, China

## Abstract

Cell therapy is a potential therapeutic approach for Parkinson's disease (PD). Mesenchymal stem cells derived from the human umbilical cord (hUC-MSCs) give priority to PD patients because of multiple advantages. The appropriate gene transduction of hUC-MSC before transplantation is a promising procedure for cell therapy. Fibroblast growth factor-20 (FGF-20) has been shown to protect dopaminergic neurons against a range of toxic insults in vitro. In this study, the hUC-MSCs were gene transduced with FGF-20, and then we transplanted them into the PD mice model. The results showed that MSC-FGF-20 treatment obviously improved the behavior of PD, accompanied by the increase of tyrosine carboxylase- (TH-) positive cell and dopamine (DA). Furtherly, immunohistochemistry disclosed that MSC-FGF-20 obviously promoted the degradation of nuclear factor-*κ*B (NF-*κ*B), a transcription factor that controls genes encoding proinflammatory cytokines, highly expressed in the nigrostriatal dopaminergic regions in PD patients. Therefore, MSC-FGF-20 has a potential for improving PD, closely related to the degradation of NF-*κ*B.

## 1. Introduction

Pathological features of Parkinson's disease (PD) are progressive loss of dopaminergic (DA) neurons selectively in the pars compacta of the substantia nigra (SN) and the presence of eosinophilic Lewy bodies in the residual DA neurons. In this condition, the amount of DA in the corpus striatum is decreased, and patients present with clinical symptoms such as tremor, rigidity, and bradykinesia [1–3]. Patients initially respond to treatment with dopaminergic-enhancing medications such as levodopa [4]. However, the effectiveness of such treatments gradually diminishes because the conversion to dopamine within the brain is increasingly disrupted by the progressive degeneration of the dopaminergic terminals.

It is known that stem cells are a type of cells that have the capability of self-renewal and multipotential differentiation, providing great hope for treatment of PD [5–7]. The mesenchymal stem cells derived from the human umbilical cord (hUC-MSCs) are more primitive and possess multiple advantages including ethical agreeableness, a less invasive procedure for isolation, low immunogenicity, high proliferation capacity, and multilineage differentiation capability [8–10]. Recently, a number of studies demonstrated that hUC-MSCs transduced by some genes could yield better effect [8–10]. In our study, the hUC-MSCs were transduced by an adenovirus carrying the fibroblast growth factor-20 (FGF-20) gene (Ad-FGF-20). In our study, the hUC-MSCs were transduced by an adenovirus carrying the fibroblast growth factor-20 (MSC-FGF-20), and then we would like to know the effect of MSC-FGF-20 on PD and compare it with that of hUC-MSC without any treatment.

FGF-20 shows preferential expression in the brain, especially substantia nigra pars compacta (SNpc) in the adults. The presence in the SNpc is especially interesting given that certain FGF-20 single nucleotide polymorphisms or haplotypes are associated with an increased risk of developing PD in some, although not all, patient studies [11, 12]. In the current study, C57BL mice, an animal model of PD, were intraperitoneally injected by 1-methyl-4-phenyl-1,2,3,6-tetrahydropyridine (MPTP). And then the behavior improvement, the level of tyrosine carboxylase (TH) and dopamine (DA), and the expression of nuclear factor-*κ*B (NF-*κ*B) were detected in PD mice after hUC-MSC and MSC-FGF-20 transplantation.

## 2. Materials and Methods

### 2.1. Reagents, Antibodies, and the Expression Vectors

Human umbilical cords were obtained from healthy women who had childbirth in our hospital. All samples obtained were provided by these women who agreed and signed consent forms. C57BL mice (6–8 weeks) were ordered from Beijing Vital River Laboratory Animal Technology Co. Collection of samples and handling animals were all approved by the Ethics Committee of Neurology Department of General Hospital of Jinan Military Region. All experiments were reviewed by the Ethics Committee of Neurology Department of General Hospital of Jinan Military Region. Adenovirus expressing green fluorescent protein gene (Ad-GFP) was provided by Beckman Medical Instruments, USA. Recombinant adenovirus carrying FGF-20 gene (Ad-FGF-20) was constructed in our lab.

Consumables such as F12 culture medium, pipettes, and 1-methyl-4-phenyl-1,2,3,6-tetrahydropyridine (MPTP) were all ordered from Sigma (USA). Fetal calf serum was ordered from HyClone Company (USA). All ELISA reagent kits were ordered from IBL (Germany). All antibodies were ordered from Abcam (England). Chromogenic substrate kit was ordered from Shanghai Ruichi Biotechnology Co., Ltd. (Shanghai, China).

### 2.2. hUC-MSC-FGF-20 Preparation

Isolation and verification of hUC-MSCs were carried out based on previously described protocols, and determination of Ad-FGF-20 optimal transduction efficiency was performed based on protocols described previously [11, 12]. The hUC-MSCs were infected by Ad-FGF-20 at multiplicity of infection (MOI) of 200 for 48 hours, and then the cells were harvested. Concentrations of immunoreactive FGF-20 in the supernatant were measured by ELISA. The FGF-20 protein was quantified by BCA assay. After being electrophoresed on 10% SDS-PAGE gel, the proteins were transferred onto FluoroTrans® W PVDF membranes (Pall, 20685) via electrophoretic transfer system (Bio-Rad). Then, the membranes were blocked with 5% skim milk in PBST for 1 hr followed by incubation with respective primary antibodies at 4°C overnight. After being thoroughly washed with PBST, the membranes were further incubated with respective horseradish peroxidase conjugated secondary antibodies. Thereafter, the protein bands were visualized with ECL-prime kit.

### 2.3. Preparation of Animal Model of PD and Behavioral Tests

C57BL mice (male, 6–8 weeks, 90) weighing approximately 20 ± 5 grams were used for preparation of animal model of PD as described previously [13]. All mice were pretrained for 3 days on a rota rod; 10 mice with uncoordinated movement were excluded before MPTP injection. The details were described as follows: 20 out of 80 mice were randomly assigned to control group, and the remaining 60 mice were injected intraperitoneally with 250 mg/kg probenecid, half an hour later, and with 25 mg/kg MPTP twice a week and continuously for 5 weeks. After giving the first three injections, mice presented with stress reactions such as severe tremor, unwillingness to move, uncoordinated gait, straightly erected tail, and spread hair, and occasionally mice had seizure-like attack; however, 2 hours later, stress reactions slowly reduced and recovered to normal condition 12 hours later; nevertheless, with increasing times of MPTP injection, although mice still showed stress reactions in response to the injection, the interval of symptoms gradually disappeared or became longer; moreover, progressively spontaneous activities were reduced; furthermore, symptoms of PD such as body tremor, arcuate curvature of back and stiffness, slow and uncoordinated movement of limbs, and slow crawling movement gradually became obvious and stable. Five weeks later, open field test and rota rod test were applied to verify whether mice model of PD was successfully made.

#### 2.3.1. Open Field Test

A 30 cm × 30 cm × 15 cm glass plastic chamber was made by us; 6 cm × 6 cm grids were scaled at the bottom of the box. The test was performed at quiet and dim environment; after mice were adapted to the environment for 10 minutes (min), the number of rearing and ambulation instances was calculated within 5 min, and means were calculated after continuous measurement was made for 5 times.

#### 2.3.2. Rota Rod Test

The mice were placed on the rotating rota rod (16 r/min) and time was counted. Latency to fall was defined as time keeping on the rota rod, that is, time for the mice falling for the first time and the number of times the mice fell from the rod within 2 min (drop frequency), which represented the ability of movement and coordination. Every mouse was monitored for 3 times with interval of 30 mins, and the average was then calculated. According to the literature [14, 15], in the open field test, successful establishment of mouse model of PD was verified as the number of rearing instances was less than 15 times/5 min and that of ambulation instances was less than 55 times/5 min, with less than 30 seconds of latency and more than 20 times/2 mins of drop frequency in the rota rod test. Five weeks later, after accidental death of mice was excluded, the total number of mice that met the criteria stated above was 50. These mice were randomly assigned to three groups based on weight: (1) model group (17), mice with MPTP and probenecid group without any treatment; (2) MSC group (17), hUC-MSCs without any treatment; (3) MSC-FGF-20 group (16), hUC-MSCs transduced by Ad-FGF-20 group. (4) Control group (C57BL mice, male, 6–8 weeks, 20) included mice without any treatment. hUC-MSCs were injected at a concentration of 10^7^/kg once a week via the tail vein. Duration of continuous treatment was 8 weeks. After treatment was finished, further observation was made continuously for 8 weeks, and then behavioral changes were examined by using the open field test and rota rod test. Six mice were sacrificed for rapid dissection of whole brains which were then fixed in 4% paraformaldehyde; paraffin embedding and sectioning were made for detection.

### 2.4. Immunohistochemistry

Paraffin sections were deparaffinized until dehydration; they were incubated in 3% H_2_O_2_ for 10 min, washed in distilled water, and soaked in phosphate buffer solution (PBS) for 5 min. Antigen retrieval was processed in citrate buffer in microwave. After natural cooling of the sections was finished, the sections were incubated in rabbit anti-TH and NF-*κ*B antibody solution (1 : 300) overnight at 4°C. They were rinsed in PBS for 3 times (5 min per time); 50 *μ*L horseradish peroxidase- (HRP-) labelled goat anti-rabbit secondary antibody was added to the sections and incubated for 30 min at 37 degrees centigrade. After the sections were rinsed with PBS for 3 times (5 min per time), 3,3′-diaminobenzidine (DAB) color staining was made and controlled under a microscope, and it was terminated under running water; after full washing, hematoxylin staining was performed: thorough dehydration and transparency and neutral gum mounting were conducted. Quantity One software was applied for imaging analysis.

### 2.5. Western Blot (WB)

The basal ganglion of 6 mice was dissected on ice and placed in lysis buffer; total tissue protein was extracted by repetitive freeze/thaw and centrifugation. Quantitative analysis of protein was made by using BCA-200 protein quantitative kit. 20 *μ*g protein taken from each sample was mixed with loading buffer and dithiothreitol (DTT) at a ratio of 8 : 10 : 2, protein was denatured by boiling for 5 min, and 12% SDS-PAGE gel was used for electrophoresis and membrane transfer. Nitrocellulose membrane was blocked with 5% skim milk for 1 hr and then incubated with rabbit anti-TH and NF-*κ*B antibody. After fully washing off uncombined antibody, the membrane was incubated with HRP anti-rabbit antibody for 1 hr; enhanced chemofluorescent staining was conducted. Quantity One software was applied for imaging analysis.

### 2.6. High Performance Liquid Chromatography (HPLC)

The striatum from 5 mice was stored in liquid nitrogen for detection. HPLC-ECD was used to detect the level of DA as described previously [16]. Samples were loaded after routine processing; maximum pressure was defined as 34.32 mPa; flow speed was 12 mL/min; working voltage was 0.80 V; sensitivity was 5 nA; different concentrations of DA standard samples were prepared; the concentration of the standard samples was set from 2 *μ*g/L to 1000 *μ*g/L to make a standard curve. The volume of standard samples and experimental samples for measurement was 150 *μ*L; measurement was made based on peak area and divided by wet weight of tissue samples; the value obtained for DA was DA content per gram of brain.

### 2.7. Statistical Analyses

Statistical analyses were performed using SPSS10.0 software. Data were presented as means ± SEM and subjected to one- or two-way analysis of variance (ANOVA), followed by either Newman-Keuls or Bonferroni's multiple-comparisons test (as a post hoc test). *p* < 0.05 was considered to indicate statistical significance. The results of immunocytochemistry and WB were analyzed by Image-Pro Plus 5.0 image analyzer (Media Cybernetics, USA). The integrated optical density (IOD) and gray values were evaluated by statistical analysis.

## 3. Results

### 3.1. hUC-MSCs Highly Expressed FGF-20 after Transduction with Ad-FGF-20

**Figure 1:**
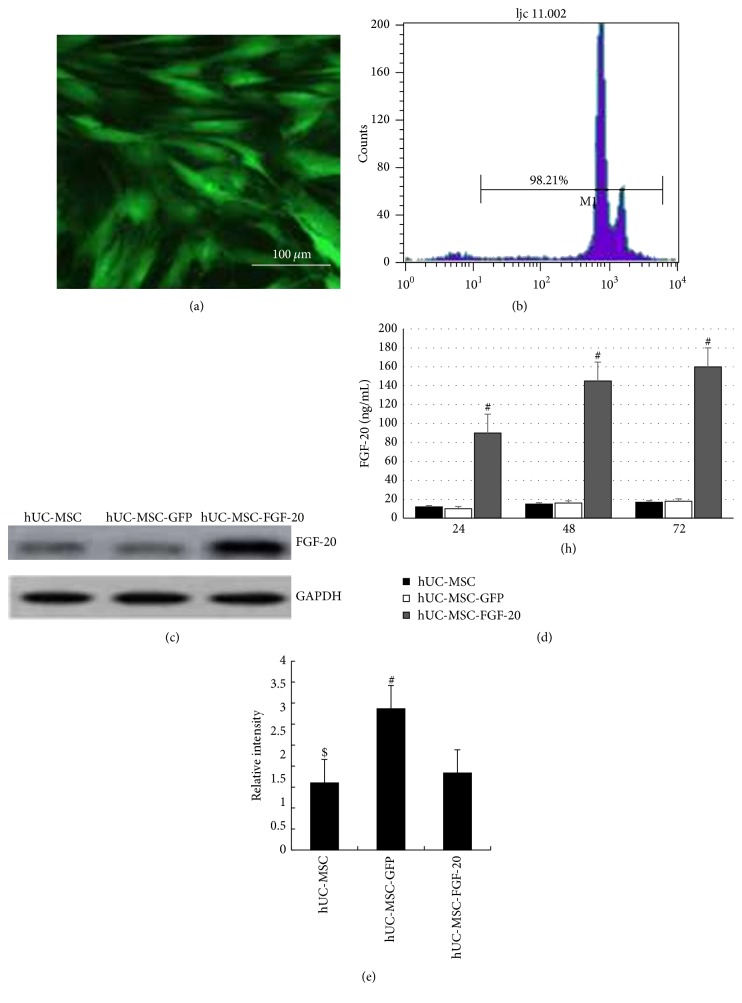
The expression of FGF-20 in transduced hUC-MSCs in vitro. (a) Expression of GFP in hUC-MSCs transduced by Ad-GFP as determined by fluorescence microscopy. (b) Flow cytometric analysis of GFP expression in UCMSC transduced by Ad-GFP. (c) Production of bioactive FGF-20 in the supernatant of hUC-MSCs. Supernatant from cultured cells was retrieved on the indicated time points, processed, and analyzed using a mouse specific FGF-20 ELISA assay. (d) Typical bands of FGF-20 and GAPDH detected by western blotting. (e) Gray intensity analysis of FGF-20. All data are presented as mean ± standard error of mean (^#^ *p* < 0.01, hUC-MSCs versus hUC-MSC-FGF-20 and UCMSC-GFP; ^$^ *p* < 0.01, hUC-MSCs versus hUC-MSC-FGF-20).

Firstly, the hUC-MSCs were transduced by Ad-GFP; the fluorescence microscopy and flow cytometry showed that greater than 90% of cells expressed GFP (Figures 1(a) and 1(b)). Ad-FGF-20 efficiently expressed high levels of transgenic FGF-20 in hUC-MSCs. As shown in Figure 1(c), FGF-20 accumulated to about 90 ng/mL in the supernatant at 24 h in Ad-FGF-20 groups. It gradually increased, peaking at 145 ng/mL at 48 d. At 72 d, concentration of FGF-20 declined slightly to approximately 160 ng/mL. In blank control and Ad-GFP groups, FGF-20 concentration remained stable at about 16 ng/mL. It is clear that FGF-20 protein level obviously increased after transduction with Ad-FGF-20 compared with hUC-MSCs alone and transduction with Ad-GFP at different time point (^#^ *p* < 0.01). Furthermore, the expression of FGF-20 was detected by WB, and the result was consistent with that of the ELISA (Figures 1(d) and 1(e)).

### 3.2. MSC-FGF-20 Improved the Behaviors of PD Mice

**Figure 2:**
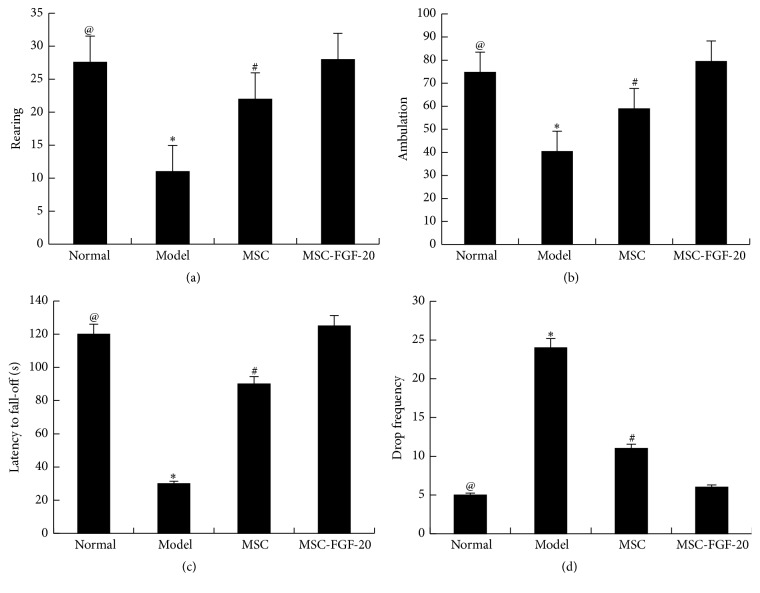
Behavioral changes in mice with PD after cell treatment. (a) Open field versus average number of rearing instances. (b) Open field versus ambulation. (c) Rota rod test versus latency to fall. (d) Rota rod test versus the drop frequency (^@^ *p* < 0.01, the normal group versus the model group; ^*∗*^ *p* < 0.01, the model group versus MSC and MSC-FGF-20 group; ^#^ *p* < 0.05, MSC group versus MSC-FGF-20 group).

Mice with PD presented with symptoms such as unsteady gait, reduced spontaneous activities, easy irritation, straightly erected tail, stiffness of the limbs, and slow and uncoordinated limb movement. These symptoms were alleviated after stem cell treatment. In the open field test, the average number of rearing instances within 5 min in the control group was 27.6, and it was 11.4 in the model group, and there was a significant difference between the two groups (^@^ *p* < 0.01), indicating that combination of MPTP with probenecid makes a successful mouse model of PD. After stem cell treatment, the number of rearing instances was 22.8 and 26.1 in the MSC group and MSC-FGF-20 group, respectively; behaviors had greater improvement in the two groups than those in the model group (^*∗*^ *p* < 0.01). Moreover, a statistically significant difference was observed between the MSC group and MSC-FGF-20 group (^#^ *p* < 0.05). Moreover, tendency of changes of ambulation was similar to those of rearing (Figures 2(a) and 2(b)).

In the rota rod test, the change of latency to fall was similar to that of rearing. In contrast, the change of drop frequency within 2 min was opposite to the change of latency to fall. Stiffness of the limbs and increased drop frequency in mice with PD were improved after stem cell treatment, and treatment effect in the MSC-FGF-20 group was even better than in the MSC group (Figures 2(c) and 2(d))

### 3.3. MSC-FGF-20 Increased the Expression of TH in Substantia Nigra and Corpus Striatum

**Figure 3:**
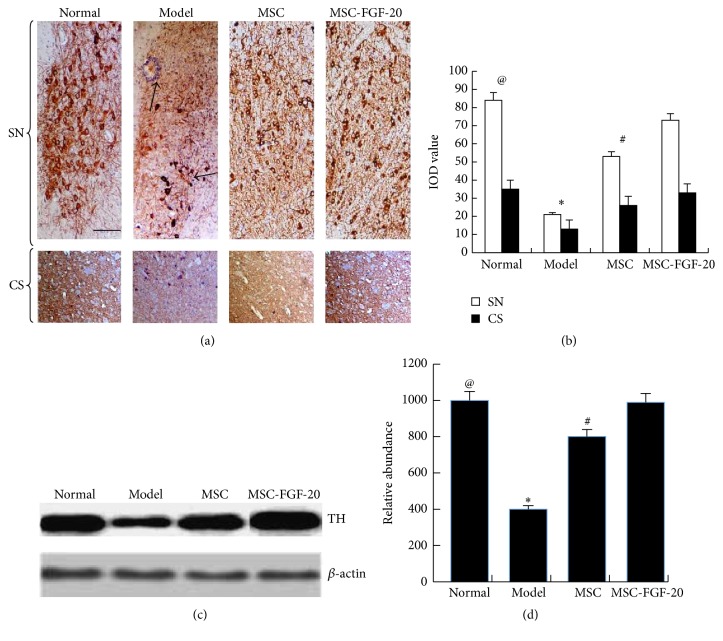
TH expressions in PD mice after cell treatment. (a, b) TH immunohistochemistry staining results showed that the number of TH-positive cells greatly decreased in the model group (scale bar = 50 *μ*m), and the cells were not neatly arranged, axons and dendrites became thinner and broken, anastomosing networks of axons became loose, and infiltration of tangled glial cells was seen (arrows). After hUC-MSC and hUC-MSC-FGF-20 treatment, TH-positive cells stained darker and the number of the cells was obviously increased. (c, d) WB results showed that tendency of TH expression changes in the corpus striatum was consistent with immunohistochemistry results (^@^ *p* < 0.01, the normal group versus the model group; ^*∗*^ *p* < 0.01, the model group versus MSC and MSC-FGF-20 group; ^#^ *p* < 0.05, MSC group versus MSC-FGF-20 group).

As shown in Figures 3(a) and 3(b), results of immunohistochemistry and image analysis showed that the number of TH-positive cells in the substantia nigra (SN) was greatly reduced in the model group when compared with mice in the normal control group. Arrangement of TH-positive cell structures was irregular. The axons and dendrites of cell became thinner and broken, and infiltration of tangled glial cells was seen (arrows). Brown-yellowish nerve fibers in the corpus striatum were obviously lighter. The imaging analysis and statistical analysis showed a statistically significant difference between the control group and the model group (^@^ *p* < 0.01). While, after treatment with hUC-MSC and MSC-FGF-20, TH-positive cells stained dark and the number of cells was markedly increased, arrangement of cellular structures appeared to be well organized, axons of neurons became longer and denser, and there was a significant difference when compared with the model group (^*∗*^ *p* < 0.01); moreover, a significant difference of the TH expression was found between the MSC group and MSC-FGF-20 group as well (^#^ *p* < 0.05). More TH-positive cells with longer and darker stained axons in the corpus striatum were observed in the MSC-FGF-20 group, when compared with the MSC group. Because the SN is too small in the mice, isolation from the basal ganglion is very difficult; in order to detect TH expression, protein was extracted from whole basal ganglion for WB (Figure 3(c)); results showed that the change of TH expression was similar to those detected by using immunohistochemistry; no repetitive descriptions were made here again; Figure 3(d) showed the results of WB image analysis.

### 3.4. MSC-FGF-20 Increased the Level of DA in the Corpus Striatum

**Figure 4:**
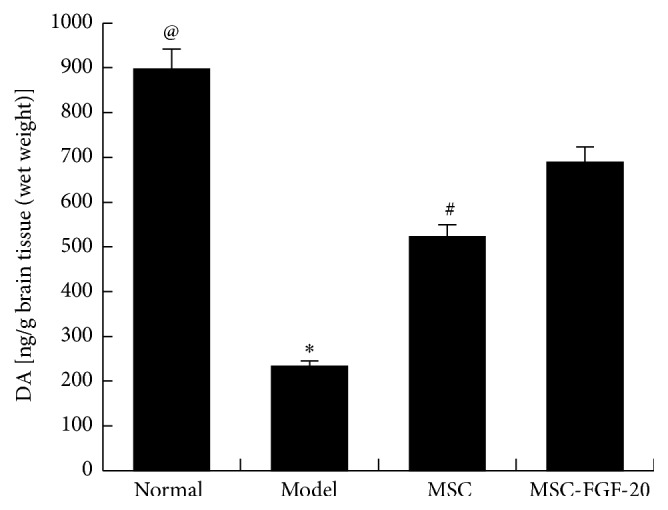
The level of DA in the corpus striatum of PD mice. DA level was greatly decreased in the model group, whereas it was obviously increased after hUC-MSC and hUC-MSC-FGF-20 treatments (^@^ *p* < 0.01, the normal group versus the model group; ^*∗*^ *p* < 0.01, the model group versus MSC and MSC-FGF-20 group; ^#^ *p* < 0.05, MSC group versus MSC-FGF-20 group).

HPLC revealed that the level of DA in the model group was significantly decreased compared with normal control group (^@^ *p* < 0.01), whereas the level was markedly increased after hUC-MSC and MSC-FGF-20 treatment (^*∗*^ *p* < 0.01), whereas DA level in the MSC-FGF-20 group was markedly higher than that in the MSC group, which showed a statistically significant difference (^#^ *p* < 0.05, Figure 4).

### 3.5. MSC-FGF-20 Reduced the Level of NF-*κ*B in the Cerebral Cortex of PD Mice

**Figure 5:**
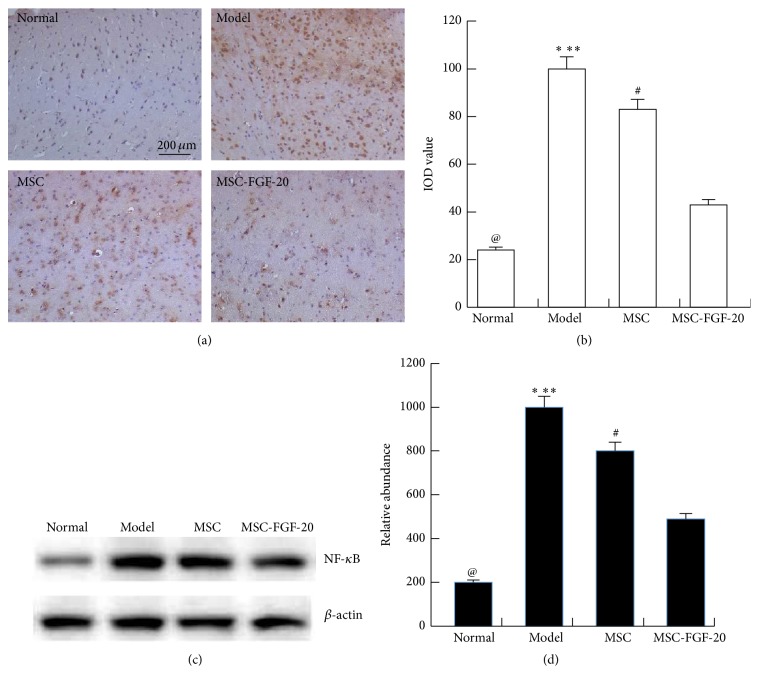
NF-*κ*B expressions in the cerebral cortex of PD mice. (a, b) The results of immunohistochemistry showed that the expression of NF-*κ*B greatly increased in the model group (scale bar = 200 *μ*m). After hUC-MSC and hUC-MSC-FGF-20 transplantation, the expression of NF-*κ*B significantly decreased in the cerebral cortex of PD mice. (c, d) WB confirmed the results of immunohistochemistry (^@^ *p* < 0.01, the normal group versus the model group; ^*∗*^ *p* < 0.05, the model group versus MSC group; ^*∗*^ *p* < 0.01, the model group versus MSC-FGF-20 group; ^#^ *p* < 0.05, MSC group versus MSC-FGF-20 group).

Furtherly, we detected the level of NF-*κ*B in the cerebral cortex by immunohistochemistry. The results showed that there were very few NF-*κ*B-positive cells in the normal group, opposite to the case in the model group (^@^ *p* < 0.01). And the level of NF-*κ*B slowly decreased in the MSC and MSC-FGF-20 group (^*∗*^ *p* < 0.05, ^*∗∗*^ *p* < 0.01), especially in the MSC-FGF-20 group (^#^ *p* < 0.05). The data of WB confirmed the immunohistochemistry results (Figure 5).

## 4. Discussion

In PD, degeneration of dopaminergic neurons in the substantia nigra pars compacta (SNpc) underlies the key motor symptoms of tremor, rigidity, bradykinesia, and postural instability. The potential of stem cells to repair PD has fascinated the researchers. Stem cell therapy has become a reliable treatment for PD because of the powerful repair functions. More and more evidence suggests that the stem cells have remarkable influence on lots of nervous system degenerative diseases after gene transduction [1–3, 17–21].

FGFs are a superfamily of proteins, most of which bind heparin and extracellular heparin sulfate proteoglycans (HSPGs) and have a homologous central core of 140 amino acids [12]. In the research report of Sleeman, FGF-20 (2.5 mg/day) for 6 days after lesion gave significant protection (w40%) against the loss of TH-positive cells in the SNpc and the loss of striatal TH immunoreactivity, accompanied by significant preservation of gross locomotion and fine motor movements and reversal of apomorphine-induced contraversive rotations. These results implied a potential neuroprotective role for FGF-20 in PD [11]. So we chose the FGF-20 as the target gene in our study.

An animal model was made to examine effect of MSC-FGF-20 on treatment of PD. MPTP is a highly selective DA neurotoxin, and it can enter the brain via the blood-brain barrier and mainly transduces neuroglial monoamine oxidase B into MPP+; DA transporter actively takes up MPP+ into DA neurons in the SN and enters into mitochondria by active transportation; MPP+ inhibits activity of mitochondria respiratory chain complex I, reduces ATP synthesis, and increases oxygen free radicals. Meanwhile, MPP+ accumulates lactic acid, makes imbalance of calcium, increases NO synthesis, and reduces synthesis of glutathione. These factors promote overproduction of all oxygen free radicals and reduce resistance of cell in response to injury of oxygen free radicals, consequently leading to necrosis of DA neurons in the SN, and a mouse model of PD is successfully made [22, 23]. However, after treatment of hUC-MSC and MSC-FGF-20, we found that MSC-FGF-20 has better treatment effects on behavioral changes; the number of DA neurons and TH expression were more than those in MSC group.

To further examine mechanisms underlying treatment effect of MSC-FGF-20, we observed the change of NF-*κ*B in the cerebral cortex of PD mice. NF-*κ*B, a transcription factor that controls genes encoding proinflammatory cytokines, adhesion molecules, chemokines, growth factors, and inducible enzymes [24–26], suggested its association with PD. Hunot et al. found a seventyfold increase in immunoreactive NF-*κ*B in proportion with dopaminergic neurons in PD brain compared with controls in a French population [27]. In the report of Yu, FGF21 ameliorates collagen-induced arthritis through modulating oxidative stress and suppressing NF-*κ*B pathway [28]. Fibroblast growth factor-inducible 14 (Fn14) is a highly inducible cytokine receptor that engages multiple intracellular signaling pathways, including NF-*κ*B and mitogen-activated protein kinase (MAPK) [29]. However, there were few reports on the relation between NF-*κ*B and FGF-20, especially in nervous system degenerative diseases. In the current study, immunohistochemistry disclosed that NF-*κ*B-positive cells slowly decreased in the MSC and MSC-FGF-20 group compared to the model group, especially in the MSC-FGF-20 group. The results showed that MSC-FGF-20 obviously promoted the degradation of NF-*κ*B.

Therefore, this study provides evidence that MSC-FGF-20 may be a promising therapeutic agent for PD patients. The interest in stem cell transplantation after gene modification for PD will remain high. This field will obviously continue to evolve, with hopes that further refinement and understanding will increase the chances that cell transplantation will someday emerge as a fruitful treatment for patients.

## Highlights

 Overall, the main highlights are as follows:

1. The effect of MSC-FGF-20 on PD treatment is much better than that of hUC-MSC without any treatment.
2. The MSC-FGF-20 transplantation obviously promoted the degradation of NF-*κ*B in the PD mice brain.
